# Metastases risk in thin cutaneous melanoma: prognostic value of clinical-pathologic characteristics and mutation profile

**DOI:** 10.18632/oncotarget.25864

**Published:** 2018-08-14

**Authors:** Antonio G. Richetta, Virginia Valentini, Federica Marraffa, Giovanni Paolino, Piera Rizzolo, Valentina Silvestri, Veronica Zelli, Anna Carbone, Cinzia Di Mattia, Stefano Calvieri, Pasquale Frascione, Pietro Donati, Laura Ottini

**Affiliations:** ^1^ Department of Internal Medicine and Medical Specialties, Unit of Dermatology, “Sapienza” University of Rome, Rome, Italy; ^2^ Department of Molecular Medicine, “Sapienza” University of Rome, Rome, Italy; ^3^ Unit of Dermatology and Cosmetology, IRCCS, University Vita-Salute San Raffaele, Milan, Italy; ^4^ Department of Oncological and Preventative Dermatological, San Gallicano Dermatological Institute, IRCCS, Rome, Italy; ^5^ Laboratory of Cutaneous Histopathology, San Gallicano Dermatologic Institute, Rome, Italy

**Keywords:** thin melanoma, metastases, prognostic factors, Breslow thickness, mutation profile

## Abstract

**Background:**

A high percentage of patients with thin melanoma (TM), defined as lesions with Breslow thickness ≤1 mm, presents excellent long-term survival, however, some patients develop metastases. Existing prognostic factors cannot reliably differentiate TM patients at risk for metastases.

**Objective:**

We aimed at characterizing the clinical-pathologic and mutation profile of metastatic and not-metastatic TM in order to distinguish lesions at risk of metastases.

**Methods:**

Clinical-pathologic characteristics were recorded for the TM cases analyzed. We used a Next Generation Sequencing (NGS) multi-gene panel to characterize TM for multiple somatic mutations.

**Results:**

A statistically significant association emerged between the presence of metastases and Breslow thickness ≥0.6 mm (*p=0.003*). None of TM with lymph-node involvement had Breslow thickness <0.6 mm. Somatic mutations were identified in 19 of 21 TM analyzed (90.5%). No mutations were observed in two not-metastatic cases with the lowest Breslow thickness (≤0.4 mm), whereas mutations in more than one gene were detected in one metastatic case with the highest Breslow thickness (1.00 mm).

**Conclusion:**

Our study indicates Breslow thickness ≥0.6 mm as a valid prognostic factor to distinguish TM at risk for metastases.

## INTRODUCTION

From a clinical and molecular standpoint, melanoma is a complex, heterogeneous and unpredictable disease. The incidence of malignant melanoma (MM) has drastically increased in the past decades [1]. Approximately 70% of new cases of MM are thin melanomas (TM), which are lesions ≤1.00 mm in Breslow thickness, and TM diagnosis are increasing around the world [2]. After local excision, thin tumors have a 10-year survival rate of 85-90% [3]. However, the high percentage of TM without recurrences is in contrast with a group that could develop recurrences and regional or distant metastases [4–7]. The TM histopathological parameters have been extensively studied in the literature and they have been associated with prognosis [8].

Several studies showed numerous parameters indicative of unfavorable prognosis for TM patients including male sex, advanced age, trunk and head/neck anatomic sites, III and IV Clark levels, presence of marked regression, absence of inflammatory infiltrate, presence of ulceration areas and high mitotic rate [9–11]. However, existing prognostic factors cannot reliably differentiate high- and low-risk TM patients. To the best of our knowledge, only a few studies have analyzed the molecular profile of TM and to date there is no molecular predictor of disease progression [12, 13]. In recent years, significant advances in the genetic field have led to the identification of specific driver mutations in melanocytic tumors [14–16]. *BRAF*, *NRAS* and *TP53* mutations are the most prevalent pathogenic alterations in melanoma [17], but key genetic changes are also identified in *CDKN2A*, *KIT*, *GNAQ* and *GNA11* genes [18–21]. Mutation frequencies of these genes change according to the different personal data and pathological features of MM [22], revealing a complex mutational pattern that could be evaluated to predict the risk of developing metastases.

In this study, we aimed at characterizing the clinical and molecular profile of metastatic and not-metastatic TM cases. First, we evaluated a possible association between the presence of metastases and the pathological features. Furthermore, we used a Next Generation Sequencing (NGS) gene panel including 15 genes, relevant in tumorigenesis and targeted therapies, with the aim to investigate multiple somatic mutations in metastatic and not-metastatic TM cases. The identification of patients with high-risk of developing metastatic disease could be potentially used in the clinical practice, in order to gain the best prognostic classification and to potentially define the best therapeutic choices.

## RESULTS

### Clinical-pathologic features and association with the presence of metastases

Twenty-one TM cases were included in this study. As shown in Table 1, seven of the 21 selected cases were metastatic and 14 were not-metastatic TM. The median age at diagnosis was 49.0 years for metastatic cases (from 37 to 74 years) and 50.5 years for not-metastatic cases (from 29 to 85 years). Four metastatic TM cases were males and three females (57.1%, 42.9%), whereas six not- metastatic TM cases were males and eight females (42.9%, 57.1%). The majority of the metastatic and not-metastatic cases (85.7% and 64.3%, respectively) were localized in trunk (specifically shoulders, abdomen or back) considered site of intermittent sun exposure. All the metastatic cases showed Clark level III, whereas among the not-metastatic cases six showed Clark level II and eight Clark level III (42.9%, 57.1%). All metastatic cases had Breslow thickness ≥0.6 mm, by contrast among not-metastatic cases seven had Breslow thickness ≤0.5 mm and seven ≥0.6 mm. A statistically significant association emerged between the presence of metastases and Breslow thickness ≥0.6 mm (*p=0.003*). The TIL grade was classified as absent in three of seven metastatic cases (42.9%) and mild to marked in all not-metastatic cases. A mitotic rate ≥1/mm^2^ was detected in three of seven metastatic cases (42.9%), by contrast all not-metastatic cases showed mitotic rate <1/mm^2^. The association between the presence of metastases and mitotic rate showed a p-value close to the statistical significance (*p=0.051*). No ulceration was observed in our TM series.

**Table 1:** Clinical-pathologic features of the 21 thin melanoma cases analyzed

Clinical-pathologic characteristics	Total cases N=21 (%)	Metastatic cases N=7 (%)	Not-Metastatic cases N=14 (%)	*p-value*
**Median age at diagnosis (range)**	49.0 (29-85)	49.0 (37-74)	50.5 (29-85)	1.000
**Sex**				
Male	10 (47.6)	4 (57.1)	6 (42.9)	
Female	11 (52.4)	3 (42.9)	8 (57.1)	0.659
**Sun exposure**^**a**^				
Chronic (head and neck)	1 (4.8)	0 (0.0)	1 (7.1)	
Continuous (lower/upper extremities)	5 (23.8)	1 (14.3)	4 (28.6)	
Intermittent (shoulders/abdomen/back)	15 (71.4)	6 (85.7)	9 (64.3)	0.741
**Clark level**				
II	6 (28.6)	0 (0.0)	6 (42.9)	
III	15 (71.4)	7 (100.0)	8 (57.1)	0.061
**Breslow thickness**^**b**^				
≤0.5 mm	7 (33.3)	0 (0.0)	7 (50.0)	
0.6-1.00 mm	14 (66.7)	7 (100.0)	7 (50.0)	**0.003**
**TIL grade** ^**c**^				
0	3 (17.6)	3 (42.9)	0 (0.0)	
1	5 (29.4)	1 (14.3)	4 (40.0)	
2	7 (41.2)	2 (28.6)	5 (50.0)	
3	2 (11.8)	1 (14.3)	1 (10.0)	0.195
**Mitotic rate/mm**^**2c**^				
<1	14 (82.4)	4 (57.1)	10 (100.0)	
≥1	3 (17.6)	3 (42.9)	0 (0.0)	0.051
**Ulceration**^**c**^				
0	17 (100.0)	7 (100.0)	10 (100.0)	
1	0 (0.0)	0 (0.0)	0 (0.0)	-

^a^According to Whiteman *et al* 2006; ^b^according to Richetta *et al* 2014; ^c^some data for each pathologic feature are not available; in bold *p-value* <0.05 considered statistically significant. Abbreviations: TIL, Tumor-infiltrating lymphocyte; N= number of cases.

### Somatic mutation profile and association with the presence of metastases

**Figure 1:**
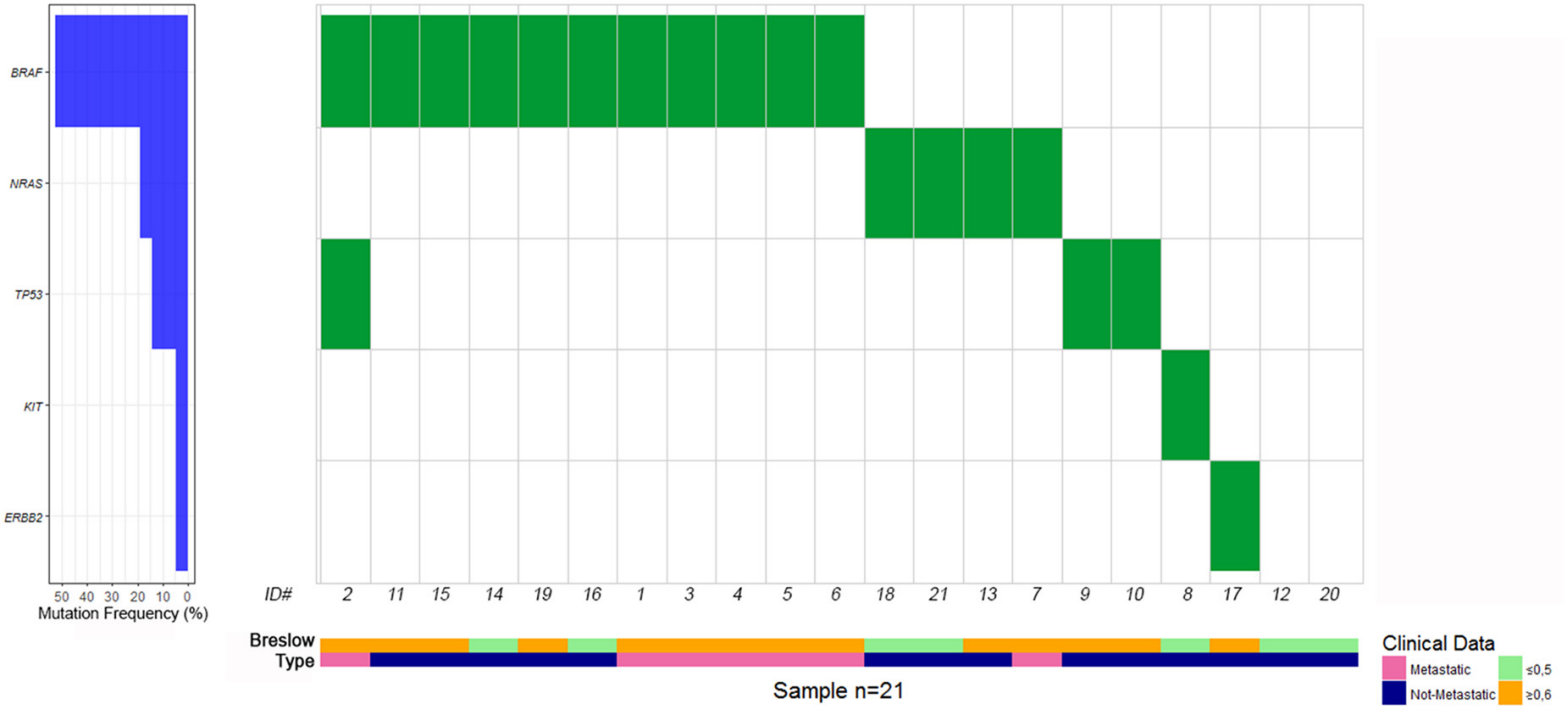
Distribution of gene mutations in metastatic and non-metastatic thin melanoma cases, labeled by pink and blue bars, respectively Thin melanoma cases with a Breslow thickness of ≤ 0.5 mm and 0.6-1 mm are showed by light green and yellow bars, respectively. To the left of the figure are the percentages of mutations identified in *BRAF, NRAS, TP53, KIT* and *ERBB2* genes.

The somatic mutation profile of the 15 most frequently mutated genes in solid tumors was screened in all 21 TM samples by TruSight Tumor 15 panel, using NGS technology. Figure 1 reports an overview of the percentages of mutations identified in *BRAF*, *NRAS*, *TP53*, *KIT*, and *ERBB2* genes, the distribution of point mutations in metastatic and non-metastatic TM cases analyzed, labeled by Breslow thickness (≤ 0.5 mm and ≥0.6 mm) and the presence or absence of metastases. We detected a total of 22 mutations in 19 of the 21 (90.5%) TM cases analyzed. All seven metastatic and 12 of 14 not-metastatic TM cases had at least one mutation in the genes analyzed (Table 2). We did not find a statistically significant difference between metastatic and not-metastatic cases and the presence of all mutations; the same results were obtained when the most frequently mutated genes (*BRAF*, *NRAS* and *TP53*) were evaluated separately (Table 2).

**Table 2:** Distribution of 21 metastatic and not-metastatic thin melanoma cases according to all gene mutations and specific mutated genes

	Total cases N=21 (%)		Metastatic cases N=7 (%)		Not-metastatic cases N=14 (%)		*p-value*
	Mutated	Wild-type	Mutated	Wild-type	Mutated	Wild-type	
**All mutations**	19 (90.5)	2 (9.5)	7 (100.0)	0 (0.0)	12 (85.7)	2 (14.3)	0.533
***BRAF***	11 (52.4)	10 (47.6)	6 (85.7)	1 (14.3)	5 (35.7)	9 (64.3)	0.063
***NRAS***	4 (19.0)	17 (81.0)	1 (14.3)	6 (85.7)	3 (21.4)	11(78.6)	1.000
***TP53***	3 (14.3)	18 (85.7)	1 (14.3)	6 (85.7)	2 (14.3)	12 (85.7)	1.000
***KIT***	1 (4.8)	20 (95.2)	0 (0.0)	7 (100.0)	1 (7.1)	13 (92.9)	-
***ERBB2***	1 (4.8)	20 (95.2)	0 (0.0)	7 (100.0)	1 (7.1)	13 (92.9)	-

Abbreviation: N= number of cases.

As shown in Table 2, *BRAF* mutations were detected in 11 cases (52.4%), *NRAS* in four (19.0%), *TP53* in three (14.3%), *KIT* and *ERBB2* in one case (4.8%) each. *BRAF* and *NRAS* were the most frequently mutated genes and their mutations were mutually exclusive. Overall, *BRAF* mutations were identified in six of seven metastatic cases (85.7%) and in five of 14 not-metastatic cases (35.7%). No mutations were identified in the other genes examined. The pathological features and the mutations identified in the series of TM cases analyzed are reported in Table 3. *BRAF* c.1799T>A (p.Val600Glu) was the most frequent mutation identified. Overall, nine of 21 (42.9%) of TM cases harbored this mutation, in particular it was detected in five of seven (71.4 %) metastatic and in four of 14 (28.6 %) not-metastatic cases. Two additional *BRAF* mutations, c.1780G>A (p.Asp594Asn) and c.1798_1799GT>CG (p.Val600Arg), were identified in two TM cases. Three *NRAS* mutations, c.182A>G (p.Gln61Arg), c.181C>A (p.Gln61Lys) and c.34G>C (p.Gly12Arg), were identified in four TM cases. Five *TP53* mutations, c.328C>T (p.Arg110Cys), c.1009C>T (p.Arg337Cys), c.1045G>T (p.Glu349Ter), c.742C>T (p.Arg248Trp) and c.11C>T (p.Pro4Leu), were detected in three TM cases. No mutations were observed in two TM cases whereas mutations in more than one gene were detected in one TM case (Table 3). Specifically, no mutations were detected in two not-metastatic cases with the lowest values of Breslow thickness (0.3 mm and 0.4 mm, respectively) whereas *BRAF* c.1799T>A (p.Val600Glu) and *TP53* c.328C>T (p.Arg110Cys) mutations were identified in one metastatic case with the highest value of Breslow thickness (1.00 mm). Associations between gene mutations and Breslow thickness (≤0.5 mm and 0.6-1 mm) distribution (Supplementary Table 1) and TIL grade distribution (Supplementary Table 2) were also tested and no statistically significant associations emerged.

**Table 3:** Pathological features and mutations identified in metastatic and not-metastatic thin melanoma cases

ID	LYMPH NODE METASTASES	BRESLOW THICKNESS	CLARK LEVEL	ULCERATION	MITOTIC RATE	TIL GRADE	BRAF (NM_004333.4, NP_004324.2)	NRAS (NM_002524.4, NP_002515.1)	TP53 (NM_000546.5, NP_000537.3)	KIT (NM_000222.2, NP_000213.1)	ERBB2 (NM_004448.2, NP_004439.2)
**#1**	YES	0.7 mm	III	NO	1/mm^2^	0	c.1799T>A, p.Val600Glu				
**#2**	YES	1.0 mm	III	NO	NO	2	c.1799T>A, p.Val600Glu		c.328C>T, p.Arg110Cys		
**#3**	YES	0.9 mm	III	NO	NO	3	c.1780G>A, p.Asp594Asn				
**#4**	YES	0.9 mm	III	NO	1/mm^2^	0	c.1799T>A, p.Val600Glu				
**#5**	YES	0.6 mm	III	NO	1/mm^2^	2	c.1799T>A, p.Val600Glu				
**#6**	YES	0.8 mm	III	NO	NO	0	c.1799T>A, p.Val600Glu				
**#7**	YES	1.0 mm	III	NO	NO	1		c.34G>C, p.Gly12Arg			
**#8**	NO	0.4 mm	II	NO	NO	1				c.2519G>A, p.Ser840Asn	
**#9**	NO	0.6 mm	III	NO	NO	3			c.1009C>T, p.Arg337Cys		
**#10**	NO	0.8 mm	III	NO	NO	2			c.1045G>T, p.Glu349Ter; c.742C>T, p.Arg248Trp; c.11C>T, p.Pro4Leu		
**#11**	NO	0.7 mm	III	NO	NO	2	c.1799T>A, p.Val600Glu				
**#12**	NO	0.3 mm	II	NO	NO	1					
**#13**	NO	0.7 mm	III	NO	NO	2		c.182A>G, p.Gln61Arg			
**#14**	NO	0.4 mm	II	NO	NO	1	c.1799T>A, p.Val600Glu				
**#15**	NO	0.6 mm	III	NO	NO	2	c.1799T>A, p.Val600Glu				
**#16**	NO	0.5 mm	III	NO	NO	1	c.1799T>A, p.Val600Glu				
**#17**	NO	0.7 mm	III	NO	NO	2					c.2521C>T, p.Leu841Phe
**#18**	NO	0.4 mm	II	NA	NA	NA		c.182A>G, p.Gln61Arg			
**#19**	NO	0.7 mm	III	NA	NA	NA	c.1798_1799delGTinsAG, p.Val600Arg				
**#20**	NO	0.4 mm	II	NA	NA	NA					
**#21**	NO	0.3 mm	II	NA	NA	NA		c.181C>A, p.Gln61Lys			

Abbreviations: TIL, Tumor-infiltrating lymphocyte; NA, not available.

## DISCUSSION

In this study we analyzed a series of TM, in order to investigate clinical-pathologic and molecular differences in metastatic and not-metastatic cases. The strategy of early detection and the greater incidence of melanomas in the last years has contributed to increase the diagnosis of TM [25]. Unfortunately, despite the excellent prognosis of TM, a small group of patients could develop metastases, fatal in 5% of the cases [26]. It is therefore of great interest to identify predictors of poor prognosis in TM patients [8]. Several authors highlighted an increased risk of progression for TM with Breslow thickness ≥0.75 or ≥0.5 mm [6, 25, 27]. Most studies analyzing TM risk progression identified ulceration and mitotic rate as main predictors [6, 27], while others indicated other risk factors [28–30]. Our results showed that the presence of lymph-node metastases was associated with Breslow thickness ≥0.6 mm (*p=0.003*). In particular, none of TM with lymph-node involvement had Breslow thickness <0.6 mm. These observations confirm Breslow thickness as the most powerful predictor of developing metastatic disease. Most studies showed that the presence of lymph-node metastases is very rare (<5%) in melanomas with Breslow thickness <0.8 mm and occur in approximately 5% to 12% of patients with primary melanomas with Breslow thickness from 0.8 to 1.0 mm [6, 31–33].

In the present study, we have also attempted to identify a different mutational pattern between metastatic and not-metastatic TM cases. We characterized multiple mutations in 15 most frequently mutated genes in solid tumors by NGS technology. The most frequent mutations identified were *BRAF* mutations (52.4%), followed by mutations in *NRAS* (19.0%) and *TP53* (14.3%). *BRAF* mutations were mainly detected in metastatic TM. This result is in line with the literature [34], confirming a more aggressive behavior for melanomas associated to *BRAF* mutations, including thin lesions.

Mutations were also found in *KIT* and *ERBB2* (4.8%). Overall, our data are in agreement with literature data showing that *BRAF* is mutated in about 50% of melanomas [14, 35, 36] and that *NRAS* is the second most frequently mutated gene [37]. In agreement with literature data [38], we also showed that the most frequent *BRAF* mutation was the c.1799T>A (p.Val600Glu). In addition, we identified two different *BRAF* mutations, c.1780 G>A (p.Asp594Asn) which results in an inactivation of *BRAF* gene [39] and c.1798_1799GT>CG (p.Val600Arg), which rarely occurs in melanoma [40]. In our series, one metastatic case showed the concurrent presence of *BRAF* and *TP53* mutations. Recent Whole Exome Sequencing (WES) study attributed a *TP53* mutation frequency of 19.0% in melanoma [14]. We identified one not-metastatic TM case with multiple *TP53* variants (c.1045G>T, p.Glu349Ter; c.742C>T, p.Arg248Trp; c.11C>T, p.Pro4Leu). To date, there are conflicting data about the role of *TP53* alterations in melanoma [41, 42] and further studies could help to clarify the role of this gene in melanoma progression. We identified the *ERBB2* c.2521C>T (p.Leu841Phe) mutation in one case. This mutation is located in the kinase domain and, to our knowledge, it has not been previously reported. In recent study no *ERBB2* mutations were found in melanoma, however the importance of *ERBB2* mutations in the kinase domain is well documented in a wide variety of human cancers [43]. Further studies are needed to clarify the role of *ERBB2* mutations in melanoma, as it may help for considering targeted therapy. We also identified the *KIT* c.2519G>A (p.Ser840Asn) mutation in one case. This mutation was found for the first time in a 2-year-old boy affected by cutaneous mastocytosis [44]. *KIT* mutation is most frequently observed in acral, mucosal and chronically sun-exposed melanomas [45, 46]. In our study, *KIT* mutation was identified in a TM located in acral site. *KIT* is another important checkpoint for the targeted therapy in melanomas, since molecules as imatinib, nilotinib and apatinib can affect melanoma cells with *KIT* mutations [44–46]. Notably, in our series only two cases were negative for the presence of mutations in the genes analyzed. Both these cases were not-metastatic cases and had low Breslow thickness (<0.4 mm). On the other hand, we detected mutations in more than one gene (*BRAF* and *TP53*) in one metastatic case with a high Breslow thickness (1.0 mm).

In conclusion, our findings support and confirm Breslow thickness as a valid prognostic factor, able to distinguish TM with a high- or low-risk of developing metastases and suggest that Breslow thickness ≥0.6 mm may be considered as a threshold value where Sentinel Lymph Node Biopsy should be discussed and considered. Future studies on a larger cohort of TM patients examined by NGS analyses are needed to provide a genetic profile that could be useful as a prognostic and predictive factor.

## MATERIALS AND METHODS

### Sample information and DNA extraction

We performed a retrospective observational study on a selected series of 21 TM cases including seven metastatic and 14 not-metastatic cases. Cases were selected to include: melanoma patients with lesions ≤1.00 mm in Breslow thickness, comparable median age at diagnosis and a comparable number of female and male individuals. The series included in the study was collected between January 2009 and December 2015 at the Department of Dermatology and Venerology, Sapienza University of Rome and at the Laboratory of Dermatopathology of the “San Gallicano” Dermatological Institute and at the Dermato-oncology and preventive Unit of San Gallicano Institute of Rome. All patients involved in the current study signed an informed consent form with a detailed description of the study protocol. The study was approved by The Local Ethical Committee (Sapienza University of Rome, Protocol 873/13). The study was performed according to the Helsinki’s declaration. All cases were characterized by the personal data (sex and age at diagnosis) and by the main clinical-pathologic features including presence of metastases, anatomic site based on sun exposure (chronic, continuous or intermittent), Clark level, Breslow thickness, mitotic rate (number of mitoses/mm^2^), ulceration status and tumor-infiltrating lymphocyte (TIL) grade based on assessment of absent, mild, moderate, or marked density (TIL grade 0-3). Genomic DNA of both tumor and normal tissue samples was extracted from 10 μm-thick microdissected formalin fixed paraffin-embedded (FFPE) sections, using QIAamp DNA FFPE tissue kit (Qiagen), according to the manufacturer’s instructions. DNA quantification was performed with Qubit dsDNA HS Assay Kit (Invitrogen), according to the instructions provided by the manufacturer.

### Target sequencing and variant classification

Genomic screening for 21 TM cases was performed with TruSight Tumor 15 panel (Illumina), including relevant regions in 15 frequently mutated genes in solid tumors *(AKT1, BRAF, EGFR, ERBB2, FOXL2, GNA11, GNAQ, KIT, KRAS, MET, NRAS, PDGFRA, PIK3CA, TP53, RET).* Briefly, genomic regions were prepared in paired-end libraries, pooled and loaded into the MiniSeq system (Illumina) for automated cluster generation, sequencing and data analysis, including variant calling. The results were annotated and filtered using Illumina Variant Studio software. Somatic mutations were identified by directly comparing the mutation profile of tumor samples with their matched normal samples. Furthermore, we filtered out somatic single-nucleotide variants (SNVs) with allele frequency <5% and those with <500 reads. *In silico* bioinformatics analyses, SIFT and Polyphen [23, 24], were used to determine the potential functional effects of identified somatic SNVs. The presence of these variants was also examined in the Catalogue of Somatic Mutations in Cancer (COSMIC). In order to direct inspect mutations, sequenced reads were visualized with the Integrative Genomics Viewer (IGV) tool, using hg19 as reference genome.

### Statistical analysis

Mann-Whitney and Fisher’s exact test were used (where appropriate) in order to evaluate the potential associations between the presence of metastases and pathological features and the potential association between the presence of metastases and the mutation profile. A p-value ≤0.05 was considered statistically significant. All statistical analyses were performed with the R software (www.r-project.org).

## SUPPLEMENTARY MATERIALS TABLES



## Abbreviations

MM: Malignant melanoma
TM: Thin Melanoma
NGS: Next Generation Sequencing
TIL: tumor-infiltrating lymphocyte
FFPE: formalin fixed paraffin-embedded
SNVs: single-nucleotide variants
COSMIC: Catalogue of Somatic Mutations in Cancer
IGV: Integrative Genomics Viewer
