# Supplementary material for: Metastases risk in thin cutaneous melanoma: prognostic value of clinical-pathologic characteristics and mutation profile
Source: Oncotarget. 2018 Aug 14;9(63):32173–81. doi: 10.18632/oncotarget.25864 (PMC6114949; doi:10.18632/oncotarget.25864)
Supplement: Supplementary file 1 [file oncotarget-09-32173-s001.pdf]

Metastases risk in thin cutaneous melanoma: prognostic value of clinical-pathologic characteristics and mutation profile

SUPPLEMENTARY MATERIALS

Supplementary Table 1: Breslow thickness distribution (≤0.5 mm and 0.6-1 mm) of 21 thin melanoma cases according to all gene mutations and specific mutated genes

	Total patients N=21 (%)		Breslow Thickness ≤0.5 mm N=7 (%)		Breslow Thickness 0.6-1 mm N=14 (%)		*p-value*
	Mutated	Wild-type	Mutated	Wild-type	Mutated	Wild-type	
**All mutations**	19 (90.5)	2 (9.5)	5 (71.4)	2 (28.6)	14 (100.0)	0 (0.0)	*0.1*
BRAF	11 (52.4)	10 (47.6)	2 (28.6)	5 (71.4)	9 (64.3)	5 (35.7)	*0.2*
NRAS	4 (19.0)	17 (81.0)	2 (28.6)	5 (71.4)	2 (14.3)	12 (85.7)	*0.6*
TP53	3 (14.3)	18 (85.7)	0 (0.0)	7 (100.0)	3 (21.4)	11 (78.6)	*0.5*
KIT	1 (4.8)	20 (95.2)	1 (14.3)	6 (85.7)	0 (0.0)	14 (100.0)	*0.3*
ERBB2	1 (4.8)	20 (95.2)	0 (0.0)	7 (100.0)	1 (7.1)	13 (92.9)	*1.0*

Abbreviations: N= number of patients.

Supplementary Table 2: TIL grade distribution of 17 thin melanoma cases according to all gene mutations and specific mutated genes

	Total patients N=17* (%)		TIL grade 0 N=3 (%)		TIL grade 1 N=5 (%)		TIL grade 2 N=7 (%)		TIL grade 3 N=2 (%)		*p-value*
	**Mutated**	**Wild-type**	**Mutated**	**Wild-type**	**Mutated**	**Wild-type**	**Mutated**	**Wild-type**	**Mutated**	**Wild-type**	
**All mutations**	16 (94.1)	1 (5.9)	3 (100.0)	0 (0.0)	4 (80.0)	1 (20.0)	7 (100.0)	0 (0.0)	2 (100.0)	0 (0.0)	*0.6*
BRAF	10 (58.8)	7 (41.2)	3 (100.0)	0 (0.0)	2 (40.0)	3 (60.0)	4 (57.1)	3 (42.9)	1 (50.0)	1 (50.0)	*0.5*
NRAS	2 (11.8)	15 (88.2)	0 (0.0)	3 (100.0)	1 (20.0)	4 (80.0)	1 (14.3)	6 (85.7)	0 (0.0)	2 (100.0)	*1.0*
TP53	3 (17.6)	14 (82.4)	0 (14.3)	3 (85.7)	0 (0.0)	5 (100.0)	2 (28.6)	5 (71.4)	1 (50.0)	1 (50.0)	*0.4*
KIT	1 (5.9)	16 (94.1)	0 (0.0)	3 (100.0)	1 (20.0)	4 (80.0)	0 (0.0)	7 (100.0)	0 (0.0)	2 (100.0)	*0.6*
ERBB2	1 (5.9)	16 (94.1)	0 (0.0)	3 (100.0)	0 (0.0)	5 (100.0)	1 (14.3)	6 (85.7)	0 (0.0)	2 (100.0)	*1.0*

Abbreviations: TIL, Tumor-infiltrating lymphocyte; N= number of patients, *TIL grade pathologic feature is not available for all thin melanoma cases.
